# Mississippi Valley Association

**Published:** 1866-05

**Authors:** 


### MISSISSIPPI VALLEY ASSOCIATION.

At the last annual meeting of the Mississippi Valley Association of Dental Surgeons, an award of two gold medals, the value of each to be one hundred dollars, was offered for the most valuable essay upon the subject of Alveolar Abscess and Diseases of the Antrum. Competition for the above awards is not to be confined to the dental specialty alone, but to all regular practitioners of medicine. It is expected that a large number of essays will come before the Committee on Awards, as the subjects given are very important ones, and the field for investigation extensive. The following are the regulations by which competitors and the committee are to be governed.

1st. That essays competing for the prizes, must be placed in the hands of the committee as early as January 1st, 1867.

2nd. That the committee have power to reject all essays presented, if regarded as unworthy of the award.

3rd. That essays be promptly disposed of by the committee, as directed by the authors.

4th. That when the committee has made the award, the approved essay be sealed up, and thus preserved till the committee report to the Association, when it and the envelope containing the author's name are to be opened.

5th. That the copyright shall belong to the Association.

6th. That the committee shall not permit any one to inspect or see the copy of the essay, till after making a report to the Association.

*Resolved*, That all dental and medical Journals be requested to publish the proposition of this Association, to award the various medals mentioned in the proceedings of this society.

Essays must be sent to Dr. Geo. Watt, Xenia, Ohio, chairman of the committee.

